# Continuous versus interrupted abdominal wall closure after emergency midline laparotomy: CONTINT: a randomized controlled trial [NCT00544583]

**DOI:** 10.1186/s13017-023-00517-4

**Published:** 2023-10-17

**Authors:** Georgios Polychronidis, Nuh N. Rahbari, Thomas Bruckner, Anja Sander, Florian Sommer, Selami Usta, Janssen Hermann, Max Benjamin Albers, Mine Sargut, Phillip Knebel, Rosa Klotz

**Affiliations:** 1 Department of General, Visceral and Transplant Surgery, Heidelberg University Hospital, Heidelberg, Germany; 2 Study Centre of the German Surgical Society (SDGC), Heidelberg, Germany; 3 Department of Surgery, Medical Faculty Mannheim, Heidelberg University, Mannheim, Germany; 4 Institute of Medical Biometry (IMBI), University of Heidelberg, Heidelberg, Germany; 5 Department of General and Visceral Surgery, Augsburg University Medical Center, Augsburg, Germany; 6 Department for General and Visceral Surgery, St. Josefs-Hospital, Dortmund, Germany; 7 Department of General, Visceral, Vascular and Thoracic Surgery, Düren Hospital, Düren, Germany; 8 Department of Visceral-, Thoracic- and Vascular Surgery, Philipps-University Marburg, Marburg, Germany; 9 Department of Surgery, Klinikum Rechts Der Isar, Technical University of Munich, Munich, Germany

**Keywords:** Incisional hernia, Abdominal wall closure, Emergency surgery

## Abstract

**Background:**

High-level evidence regarding the technique of abdominal wall closure for patients undergoing emergency midline laparotomy is sparse. Therefore, we conducted a randomized controlled trial (RCT) to evaluate the efficacy and safety of two commonly applied abdominal wall closure strategies after primary emergency midline laparotomy.

**Methods/design:**

CONTINT was a multi-center pragmatic open-label exploratory randomized controlled parallel trial. Two different abdominal wall closure strategies in patients undergoing primary midline laparotomy for an emergency surgical intervention with a suspected septic focus in the abdominal cavity were compared: the continuous, all-layer suture and the interrupted suture technique. The primary composite endpoint was burst abdomen within 30 days after surgery or incisional hernia within 12 months. As reliable data on this composite primary endpoint were not available for patients undergoing emergency surgery, it was planned to initially recruit 80 patients and conduct an interim analysis after these had completed the 12 months follow-up.

**Results:**

From August 31, 2009, to June 28, 2012, 124 patients were randomized of whom 119 underwent surgery and were analyzed according to the intention-to-treat (ITT) principal. The primary composite endpoint did not differ between the continuous suture (C: 27.1%) and the interrupted suture group (I: 30.0%). None of the individual components of the primary endpoint (reoperation due to burst abdomen after 30 days (C: 13.5%, I: 15.1%) and reoperation due to incisional hernia (C: 3.0%, I:11.1%)) differed between groups. Time needed for fascial closure was longer in the interrupted suture group (C: 12.8 ± 4.5 min, I: 17.4 ± 6.1 min). BMI was associated with burst abdomen during the first 30 days with an OR of 1.17 (95% CI 1.04–1.32).

**Conclusion:**

This RCT showed no difference between continuous suture with slowly absorbable suture versus interrupted rapidly absorbable sutures after primary emergency midline laparotomy in rates of postoperative burst abdomen and incisional hernia after one year. However, the trial was stopped after the interim analysis due to futility as there was no chance to show superiority of one suture technique.

**Supplementary Information:**

The online version contains supplementary material available at 10.1186/s13017-023-00517-4.

## Background

Although the minimally invasive approach to the abdominal cavity is becoming increasingly common, midline laparotomy is frequently applied in the emergency setting due to the high accessibility and speed [1]. Post-laparotomy complications, especially related to the abdominal wall such as burst abdomen and incisional hernias, are prevalent.

After elective surgery, the incidence of early fascial dehiscence (burst abdomen) ranges from 0.4 to 3.5%, depending upon the type of surgery performed [2–4]. Fascial dehiscence increases hospital length of stay (LOS), is associated with higher mortality and emergency surgery is frequently reported as a risk factor for its occurrence [5]. Incisional hernias after midline incision present with a frequency of 3.0 to 20.0% even up to 64 months after the initial operation [2, 6–9], reduce quality of life and create the risk of incarceration or strangulation with a need for emergency surgery. In emergency surgery reported rates of fascial dehiscence range from 2.4 to 23.5% and incisional hernias are reported in 11.2–22.0% [10–12].

While surgical site infection prevention and respecting anatomical structures during abdominal wall closure are widely approved concepts, various different wall closure techniques are applied [13, 14]. The surgical strategy of abdominal wall closure, that is, the combination of suture technique and material, is highly relevant for prevention of fascia dehiscence and is the main factor directly controllable by the surgeon.

Numerous randomized controlled trials (RCTs) [15–19] as well as meta-analyses [20–23] have addressed the issue of optimal fascia closure in elective laparotomies, and after 2015 it was established that a continuous suture using the “small-bites” technique is the ideal strategy to reduce incisional hernias [24]. However, RCTs regarding abdominal wall closure in the emergency setting are sparse and as a result of the low level of available evidence, abdominal fascia closure after emergency laparotomy is performed according to the surgeon’s individual preference [10, 25, 26].

We therefore conducted an RCT to compare the two most established strategies for abdominal wall closure—continuous and interrupted—to determine an advantage of either strategy regarding the development of burst abdomen within 30 days or incisional hernia after one year.

## Methods

### Trial design

The study protocol was approved by the ethics committee of the University of Heidelberg (S-206/2007), the trial was internationally registered (NCT 00544583, October 16, 2007), and the study protocol was published to ensure transparency of the design and analysis procedures[27]. CONTINT was initiated as a single center RCT in the Department of General, Visceral and Transplantation Surgery of the University of Heidelberg. The number of trial institutions was extended, and the trial protocol was amended to a total of 12 surgical sites on August 31, 2009. The multi-center, pragmatic, intra-operatively randomized, controlled, two-group trial, was managed and monitored by the Study Centre of the German Surgical Society (SDGC) and analyzed by the Institute of Medical Biometry (IMBI), University of Heidelberg. Reporting of the trial adheres to the recommendations of the updated and extended Consolidated Standards of Reporting Trials (CONSORT) Statement.

### Participants

The inclusion/exclusion criteria, trial interventions, randomization process, definitions of endpoints and follow-up have been previously described [27]. In short, patients ≥ 18 years of age in need of an emergency midline laparotomy because of a septic focus (e.g., perforated stomach ulcer, perforated diverticulitis) with written informed consent and a life expectancy of at least 12 months were eligible for participation. While patients with previous laparotomy and a planned second look operation were excluded, patients with previous minor laparoscopic surgery (apart from colon surgery) were included.

### Randomization and blinding

Randomization was performed in permuted blocks using sealed opaque envelopes prepared by the IMBI. Intraoperatively, randomization took place after successful source control and abdominal lavage and before the closing of the abdominal wall.

The outcome assessors of the CONTINT trial were blinded to the patients’ trial intervention. The primary endpoint had to be assessed by a board-certified surgeon familiar with the examination of the abdominal wall and at least six months training in ultrasound.

### Interventions

For both groups, the distance between the stitches had to be no more than 1.5 cm and the distance from the edge of the fascia had to be at least 2 cm. For patients in the continuous suture group the abdomen was closed by a continuous, all-layer suture using two Monoplus^â^ USP 1 (0.4 mm diameter), 150 cm loops, which are made of a slowly absorbable monofilament material. Two sutures started at the wound edges, had to be anchored cranial and caudal of the incision and had to overlap in the middle for at least 2 cm. In the interrupted suture group Vicryl^©^ USP 2 (0.5mm diameter), 45 cm absorbable sutures were used starting from the cranial end to the middle of the incision and then from the caudal pole also with anchoring of stitches cranial and caudal of the incision. The sutures were tied only after all the stitches had been performed. The subcutaneous tissue was not sutured, and no subcutaneous drainage was applied while the skin closure was performed with clips. Antibiotic prophylaxis and therapy were carried out according to local standards. Electric cautery was used to cut skin, the subcutaneous tissue, the abdominal fascia, and the peritoneum, carefully avoiding damage to the umbilicus. Opening of the peritoneum was performed with scissors. Abdominal drains were placed at the end of surgery.

### Outcomes

The composite primary endpoint was the presence of burst abdomen after 30 days or incisional hernia after 12 months. Burst abdomen was defined as postoperatively missing continuity of the abdominal fascia in combination with a wound dehiscence and/or a consecutive redo surgery due to fascial dehiscence occurring up to day 30 after surgery. Incisional hernia was assessed by physical examination and abdominal ultrasound 12 months postoperatively and was defined as a fascia gap and a protruding hernia sac on ultrasound or with a clinical examination consistent with a hernia. In cases of hernia confirmed by a surgical intervention within 12 months after the index operation no ultrasound examination was mandatory.

Secondary outcome measures included length of skin and fascia incision, time needed for fascial closure, frequency of re-operation due to burst abdomen and due to any cause, frequency of abdominal re-interventions, postoperative pulmonary infection, duration of artificial respiration and postoperative hemodialysis. Furthermore, frequency of wound infection, duration of vacuum therapy and wound healing, time to first bowel movement, duration of abdominal drainage via intraoperatively placed drains and duration of closed abdominal lavage were evaluated. Finally, we assessed LOS, duration of intensive care unit stay, quality of life (by using the standardized form (SF 36)) and overall mortality.

### Statistical analysis

As empirical data for the primary endpoint were not available in the planning stage, the overall rate and treatment effect regarding the primary endpoint were uncertain. Consequently, a sample size calculation was highly uncertain, and thus, the study was performed with an adaptive interim analysis. This design allowed for early stopping of the trial or, if continued, modification of design characteristics—such as recalculation of the sample size—under control of the global type I error rate. The adaptive interim analysis was planned beforehand to take place after the completion of the 12 months follow-up for 80 evaluable patients [28]. The null hypothesis was assessed by testing the effect of the wall closure procedure in a logistic regression model that takes into account the covariates “wall closure procedure” (continuous / interrupted), BMI (values as measured on the original scale), and age (values as measured on the original scale). The global one-sided type I error rate was set at $\alpha = 0.025$ and the boundary for the one-sided p value for accepting the null-hypothesis within the interim analysis was $\alpha_{0} = 0.40$. This approach is equivalent to two-sided testing of $H_{0}$ and assures control of the global two-sided type I error rate of 0.05 within the chosen adaptive two-stage design.

Data were described using appropriate measures of location. Due to missing documentation of endpoints and as per initial protocol, patients with missing endpoint documentation on visit 5 (12 months) or more than one occurrence of missing endpoint documentation were documented as missing while patients with at least one endpoint confirmation during the follow-up period (i.e., burst abdomen or incisional hernia) were categorized as positive cases; all others were treated as negative cases. A detailed depiction of this ruling is presented in Additional file 1: Figure S1. If a patient discontinued from the study prematurely, missing data with respect to the primary outcome variable were replaced by the Imputed Case Analysis- reasons (ICA-r) method described by Higgins et al.

The primary endpoint was investigated in a logistic regression model taking into account the group (continuous / interrupted), as well as the covariates BMI (values as measured on the original scale), and age (values as measured on the original scale). For the evaluation of the serious adverse events all available data in the database were considered. Calculations were performed using SAS software (version 9.4; SAS Institute, Inc., Cary, NC, USA). In the case of missing data in secondary endpoints, patients were excluded from statistical analysis of the outcome measure concerned. Due to the explorative nature of the trial all reported p values have to be treated as descriptive statistics without confirmatory value.

## Results

### Trial flow

**Fig. 1:**
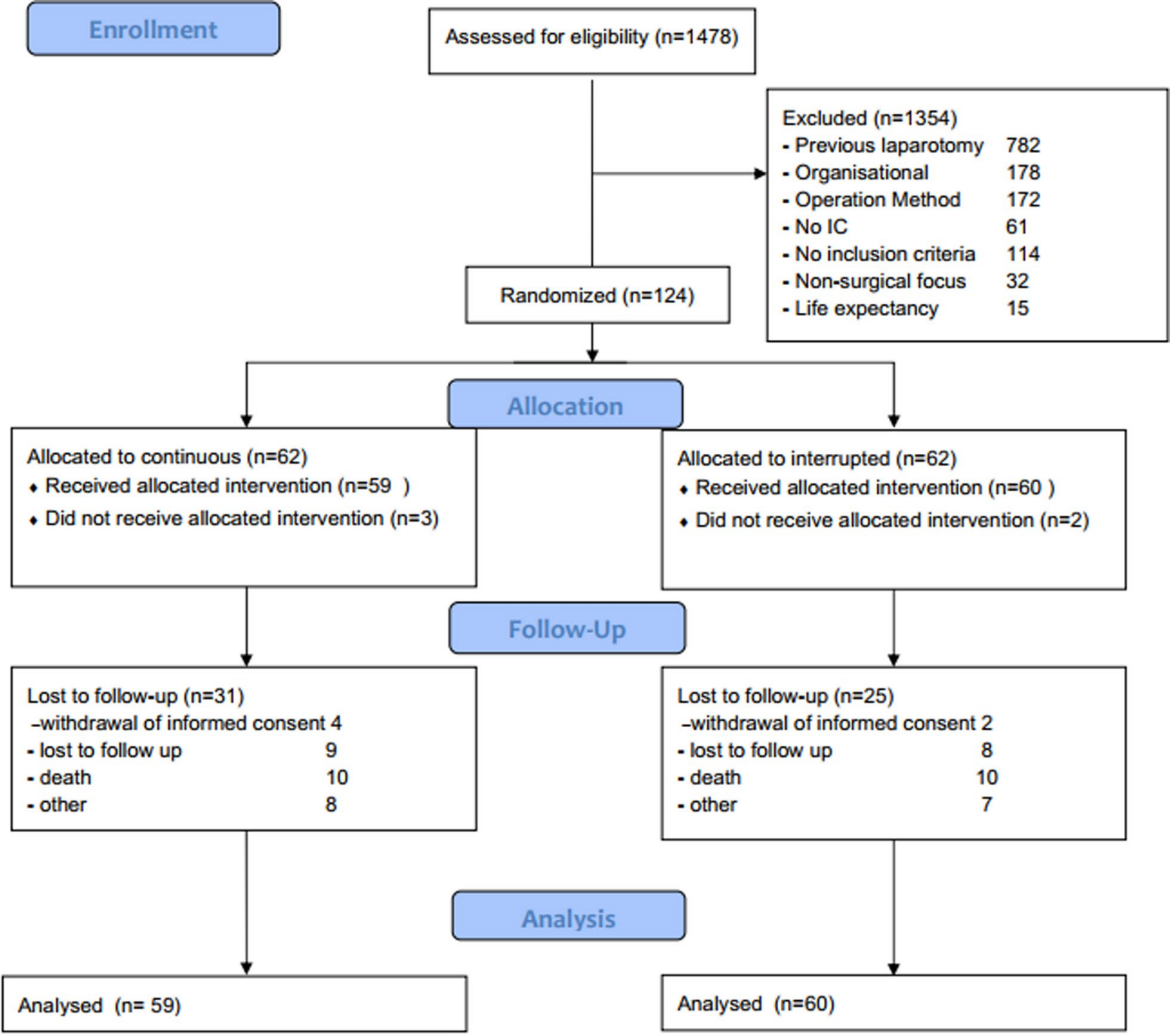
CONSORT diagram of trial conduct

Between August 12, 2009, and February 2, 2014, out of 1478 consecutively screened patients a total of 124 patients were randomly assigned to the intervention or the control group (Fig. 1). 4 patients in the continuous suture group and 2 patients in the interrupted suture group did not receive the planned intervention. As data from 31 and 25 patients were not available one-year postoperatively due to death (C: *n* = 10, I: *n* = 10), lost to follow up (C: *n* = 9, I: *n* = 8), withdrawal of informed consent (C: *n* = 4, I: *n* = 2) and other (C: *n* = 8, I: *n* = 7), 32 and 37 patients reached the one-year follow-up, respectively. However, using ICA-r imputation the total number of patients in the final analysis dataset regarding the primary endpoint was 59 for the continuous suture and 60 for the interrupted suture group. An interim analysis had been planned at 80 patients with full follow-up. Considering the high loss to follow-up rate and the results of the interim analysis for the intention-to-treat population which didn’t demonstrate any clear clinical advantage it was deemed by the study evaluation board to not perform further recruitment.

### Patients’ baseline characteristics

The baseline characteristics of the trial participants are presented in Table 1 and were comparable between the groups especially in terms of chronic disease and morbidity.

**Table 1:** Baseline characteristics

	Continuous suture (*N* = 59)	Interrupted suture (*N* = 60)
Sex		
Male	39 (66.1%)	36 (60.0%)
Female	20 (33.9%)	24 (40.0%)
Age (years)	62.1 ± 16.4	65.1 ± 13.8
Body mass index (kg/m^2^)	26.5 ± 5.9	27.3 ± 9.2
ASA classification		
I (normal healthy patient)	4 (6.8%)	4 (6.7%)
II (mild systemic disease)	19 (32.2%)	22 (36.7%)
III (severe systemic disease)	34 (57.6%)	26 (43.3%)
IV (constant threat to life)	2 (3.4%)	7 (11.7%)
V (moribund state)	0 (0.0%)	1 (1.7%)
Reason for operation		
Suspected diverticular abscess with perforation	12 (20.3%)	19 (31.7%)
Suspected stomach/duodenal perforation	20 (33.9%)	13 (21.7%)
Suspected ischemia	7 (11.9%)	9 (15.0%)
Other	20 (33.9%)	19 (31.7%)
Comorbidities		
Diabetes mellitus	8 (13.6%)	6 (10.2%)
Chronic pulmonary disease	11 (18.6%)	7 (11.7%)
Current immunosuppressive therapy	7 (11.9%)	4 (6.7%)
Current smoker	21 (35.6%)	14 (23.3%)
Current smoker years	26.2 ± 12.1	38.9 ± 12.1
Previous smoker	11 (18.6%)	16 (26.7%)
Ongoing malignancy at time of surgery	8 (13.6%)	5 (8.3%)
Previous minor abdominal incisions	11 (18.6%)	15 (25.0%)

*ASA* American Society of Anesthesiologists

Data are presented as means (with standard deviations) or numbers of patients (with percentages)

### Surgical and perioperative data

The intraoperative findings and surgical procedure characteristics were comparable between the two groups (Additional file 1: Table S1). The mean duration of hospital stay was similar between groups (C: 16.4 ± 13.3 days, I: 17.1 ± 12.1 days). The frequency of laparostomy was 7.3%, leading to the exclusion of these patients for further evaluation of hernia/dehiscence.

### Primary endpoint

The composite primary endpoint rates of burst abdomen after 30 days or incisional hernia after 12 months did not differ between the continuous and the interrupted group (C: *n* = 16 (27.1%), I: *n* = 18 (30.0%)) (Table 2). In each group one patient presented with bulging hernia sac at day 30 (*p* = 0.73). During the trial period a total of 6 patients presented with burst abdomen and needed re-operation (C: *n* = 4 (8.9%), I: *n* = 2 (4.3%), *p* = 0.38). The incidence of a palpable gap did not differ significantly (C: *n* = 1 (4.5%), I: *n* = 4 (13.8%), *p* = 0.27). A logistic regression analysis was undertaken for the primary endpoint based on previous findings that BMI and age played a major role on the appearance of fascial dehiscence. In the analysis of both the ITT and the PP set, age did not have a significant association with the primary endpoint. On the contrary, BMI showed an OR of 1.17 (95% CI: 1.04–1.32).

**Table 2:** Primary endpoint results

	Continuous suture (*N* = 59)	Interrupted suture (*N* = 60)	*p*-value*
Burst abdomen until day 30 or incisional hernia until month 12 (ITT)	8/32 (25.0%)	11/37 (29.7%)	0.66
Composite endpoint with ICA-r imputation (ITT)	16/59 (27.1%)	18/60 (30.0%)	0.73
Composite Endpoint (PP)	8/32 (25.0%)	11/37 (29.7%)	0.66
Composite endpoint with ICA-r imputation (PP)	16/59 (27.1%)	18/60 (30.0%)	0.73
Primary endpoint (ITT)	Ref = 1	OR: 1.04 (95% CI: 0.31- 3.43)	0.95
Primary endpoint (PP)	Ref = 1	OR: 1.19 (95% CI: 0.28- 5.04)	0.82

*ITT* intention-to-treat, *PP* per protocol, * p values are reported according to χ2 test

### Secondary endpoints

The secondary endpoints are presented in Table 3. The overall mortality (irrespective of cause) was *n* = 20 (16.9%) and presented with no difference between groups (C: *n* = 10 (16.9%), I: *n* = 10 (16.9), *p* = 1.00). The time needed for fascial closure was significantly shorter in the continuous than in the interrupted group (C: 12.8 ± 4.5 min versus 17.4 ± 6.1 min; *p* < 0.001). A total of 42.4% of patients developed a wound infection during their trial participation, involving mainly superficial tissue.

**Table 3:** Evaluation of hernia/ burst abdomen and follow-up data (3 months and 1 year)

	Continuous suture (*N* = 59)	Interrupted suture (*N* = 60)	*p* value
Bulging hernial sac on day 30 (telephone interview)	1 (2.3%)	1 (2.2%)	
Unclear	3 (6.8%)	1 (2.2%)	0.56
Missing	15	14	
Palpable fascia gap on day 30 (telephone interview)	1 (2.3%)	2 (4.3%)	
Unclear	3 (6.8%)	1 (2.2%)	0.50
Missing	15	14	
Bulging hernial sac at 12 months (clinical examination)	3 (13.6%)	2 (6.9%)	0.42
Missing	37	31	
Palpable fascia gap at 12 months (clinical examination)	1 (4.5%)	4 (13.8%)	0.27
Missing	37	31	
Bulging hernial sac at 12 months (ultrasound examination)	1 (6.7%)	0 (0.0%)	0.21
Missing	44	37	
Palpable fascia gap at 12 months (ultrasound examination)	0 (0.0%)	4 (17.4%)	0.09
Missing	44	37	
Re-operation due to burst abdomen	4 (8.9%)	2 (4.3%)	0.38
Re-operation due to hernia	1 (3.0%)	4 (11.1%)	0.20
Completed the trial regularly according to the protocol	28 (47.5%)	32 (54.2%)	0.46
Reason for early trial termination			
Withdrawal of informed consent	4 (12.9%)	2 (7.4%)	0.90
Lost to follow up	9 (29.0%)	8 (29.6%)	
Death	10 (32.3%)	10 (37.0%)	
Other	8 (25.8%)	7 (25.9%)	

Data are presented as means (with standard deviations) or numbers of patients (with percentages), *p* values are reported according to *χ*^2^ test for categorical variables and *t* test for continuous variables

The safety analysis in the as-treated population yielded similar rates of patients with at least 1 SAE for the 2 groups (C: *n* = 28 (46.7%), I: *n* = 31 (52.5%), *p* = 0.52). There were no marked differences in the frequency, severity, and outcome of SAEs or regarding their relationship to the trial intervention (Table 4).

**Table 4:** Secondary endpoints

	Continuous suture group (*N* = 59)	Interrupted suture group (*N* = 60)	*p* value
Mortality/death due to any cause—yes	10 (16.9%)	10 (16.9%)	1.00
Length of skin incision [cm]	20.9 ± 5.3	22.5 ± 5.9	0.17 *^2^
Length of fascial incision [cm]	22.7 ± 5.3	24.6 ± 6.5	0.11
Time needed for fascial closure [min]	12.8 ± 4.5	17.4 ± 6.1	< 0.001
Re-operation due to other reason than hernia/burst abdomen	12 (24.0%)	13 (25.0%)	0.91
Puncture of the abdominal cavity for any reason	0 (0.0%)	3 (5.4%)	0.09
Postoperative pulmonary infection	7 (14.9%)	4 (8.5%)	0.34
Duration of artificial respiration [days]	2.5 ± 9.0	1.7 ± 4.2	0.56
Duration of postoperative hemodialysis [days]	0.9 ± 3.9	0.9 ± 2.7	0.97
Wound infection	17 (34.7%)	25 (50.0%)	0.12
Duration of vacuum therapy [days]	1.4 ± 5.1	1.5 ± 5.2	0.99
Duration of wound healing in patients with secondary wound healing [days]	45.5 ± 32.9	37.7 ± 18.2	0.57
Time to first bowel movement [days]	2.6 ± 1.5	2.7 ± 1.3	0.77
Duration of abdominal drainage [days]	6.0 ± 4.2	6.9 ± 6.8	0.46
Duration of closed abdominal lavage [days]	0.0 ± 0.0	0.4 ± 2.4	0.25
Postoperative duration of hospital stay [days]	16.4 ± 13.4	17.4 ± 12.9	0.68
Postoperative duration of intensive care unit stays [days]	5.8 ± 11.7	5.6 ± 8.8	0.95

Data are means (with standard deviations) or numbers of patients (with percentages), *p* values are reported according to *χ*^2^ test for categorical variables and *t* test for continuous variables

Quality of life (QoL) was evaluated 30 days and 12 months after emergency laparotomy using the SF36. Patients reported deterioration in QoL at 30 days after surgery and an improvement was seen at 12 months postoperatively although more than 17% of the patients reported much lower QoL compared to preoperative levels. All other secondary endpoints showed no significant change (Additional file 1: Tables S2, S3).

## Discussion

CONTINT is the first multicenter RCT comparing continuous and interrupted abdominal wall closure with the composite primary endpoint incidence of incisional hernia and burst abdomen after emergency midline laparotomy. No significant difference between continuous and interrupted closure was found regarding the primary endpoint and any other postoperative complications. Length of hospital, length of intensive care unit stay and quality of life 1 and 12 months after surgery were comparable between groups. However, time needed for abdominal wall closure was significantly longer in the interrupted suture group than in the continuous group.

The rate of incisional hernia or burst abdomen in our trial is considerably higher than the rates reported in elective surgery trials [29–33]. Previously reported difference in favor of interrupted closure was not corroborated by our findings. The INLINE systematic review and meta-analysis which compared both elective and emergency surgery patients had shown a 11.3% incisional hernia incidence for the continuous closure and 7.9% for interrupted whereas we found a 27.1% and 30% occurrence of the composite endpoint, respectively [32]. Although the studies included in this systematic review had similar length of follow-up (12 months), they did not apply the same rigorous criteria as CONTINT with clinical and ultrasound examination as part of the evaluation. A short follow-up period in previous trials is another reasonable explanation for this discrepancy as shown in the publication of the 3-year follow-up of the INSECT trial participants, in which the rate of incisional hernia increased from 12.3% after 1 year to 23.2% after 3 years [30].

An analogous US trial comparing slowly absorbable interrupted polydioxanone sutures to a continuous suture in the emergency setting showed far lower rate of early fascial dehiscence (not reporting reoperations due to burst abdomen), but similar rates of incisional hernias after 1 year (13.5% for interrupted and 22.0% for continuous). Such high rates remain commonplace after emergency midline incisions, contrary to the findings of large RCTs in elective surgeries (e.g., 12.6% in the INSECT and 21% in the STITCH trial) [34–36]. The 6.6% rate of reoperation due to burst abdomen in our ITT dataset is in accordance with contemporary data not using the small-bites technique which was recommended by the EHS in 2015 [26]. In a single-center one-arm trial compared to historical data a reduction was seen in postoperative fascial dehiscence from 6.6% to 3.8% after converting the institutional standard to this technique [37].

In our analysis BMI was a risk factor for the presence of incisional hernia or burst abdomen thus confirming findings of previous retrospective studies [38]. This was not the case with patients’ age [8, 9]. Other potential risk factors such as COPD, anemia, and catecholamine-therapy were not further analyzed.

Abdominal wall closure happens at the end of the operation, and in the emergency setting this can mean that patients need to be transferred to intensive care expeditiously to stabilize the cardiac, respiratory and metabolic situation. Thus, the time needed for the abdominal wall suture is of importance. Considering both approaches, continuous suturing exhibited significantly faster speed compared to interrupted sutures. This aspect could hold particular significance during emergency laparotomy closure, where timing plays a critical role.

We reported a 16.9% mortality in both groups, a finding comparable to those of a retrospective cohort study of the ACS-NSQIP database during the same time [39] reporting a mortality rate of 12.5% during the first postoperative month. These data highlight the increased mortality associated with emergency surgery both in the US and Europe. In a subgroup analysis, we found that ongoing malignancy at baseline and preoperative pneumonia were significantly more prevalent in the group of patients lost during follow-up. Our findings also suggest that the influence of an emergency laparotomy on the quality of life is not negligible, concurring with the existing literature [40].

Our study has some considerable strengths. Primarily, the use of the composite endpoint deems our findings clinically relevant. Further, the rigorous evaluation of hernia minimizes the risk of reporting bias. Several limitations must also be considered. The most important shortcoming of this trial is the high number of patients lost to follow-up mainly due to high postoperative mortality (16.9%) which along with patients’ consent withdrawal during follow-up (10.3% in the current study) have been highlighted by similar studies [10]. Moreover, recruitment of patients was complicated by the emergency setting where time and resources are often limited. Furthermore, the large screened to randomized patient ratio implies a high risk of selection bias and limits the external validity of our results. Adding to that, the follow-up time of 1 year for incisional hernia might not be absolutely representative of the incisional hernia rates occurring after emergency midline laparotomy [41, 42]. More than half of all screened patients fulfilled the exclusion criterion of previous laparotomy. However, this exclusion criterion had been chosen to achieve a homogenous patient population regarding risk of hernia occurrence. Finally, it can be hypothesized that the lack of subcutaneous tissue closure could cause higher wound infection rates and burst abdomen in certain patients [43].

These challenges should be considered when planning for an RCT in a similar setting. Heterogeneous definitions of emergency surgery and the complexity of cases in tertiary centers might induce survivorship bias to a trial but adding regional or rural centers with solid clinical trial organization can balance those. With regard to enrollment, trials can use a stratified design to minimize selection bias. Finally, adding patients’ groups and other stakeholders into clinical trial design could reduce the rate of lost to follow-up due to withdrawal of consent.

## Conclusions

Abdominal wall closure is an important surgical step after emergency laparotomy, as it is frequently associated with complications. Evidence regarding differences in rates of postoperative burst abdomen or incisional hernia depending on the surgical technique of abdominal wall closure is sparse. After a careful appraisal of the available data no evidence for or against either of the two abdominal techniques (continuous versus interrupted) as prophylaxis of fascia dehiscence after emergency laparotomy was generated. However, the impact of our results is reduced by the high drop-out rate during follow-up. Until further high-quality RCTs are published both techniques of abdominal wall closure should be considered adequate alternatives in the emergency setting.

### Supplementary Information

**Additional file 1: Table S1.** Surgical and perioperative data.** Table S2.** SF36 Data 30 days after emergency laparotomy (Visit 4).** Table S3.** SF36 Data 12 months after emergency laparotomy Visit 5 (FAS).** Fig. S1.** Handling of missing follow up visits.

## Data Availability

The datasets used and/or analyzed during the current study are available from the corresponding author on reasonable request.

## Abbreviations

ASA: American Society of Anesthesiologists;
CONSORT: Consolidated Standards of Reporting Trials
IMBI: Institute of Medical Biometry
ITT: Intention to treat
RCT: Randomized controlled trial
KSC: Clinical Study Center
LOS: Length of hospital stay
POD: Postoperative day
PP: Per protocol
QoL: Quality of life
SAE: Serious adverse event
SF-36: 36-Item Short Form Survey
