# Supplementary material for: Continuous versus interrupted abdominal wall closure after emergency midline laparotomy: CONTINT: a randomized controlled trial [NCT00544583]
Source: World J Emerg Surg. 2023 Oct 17;18:51. doi: 10.1186/s13017-023-00517-4 (PMC10583371; doi:10.1186/s13017-023-00517-4)
Supplement: Supplementary file 1 — Additional file 1: Table S1. Surgical and perioperative data. Table S2. SF36 Data 30 days after emergency laparotomy (Visit 4). Table S3. SF36 Data 12 months after emergency laparotomy Visit 5 (FAS). Fig. S1. Handling of missing follow up visits. [file 13017_2023_517_MOESM1_ESM.docx]

**Supplementary Materials**

| Table S1: Surgical and perioperative data | | | |
| --- | --- | --- | --- |
|  | Continuous suture  (N=59) | Interrupted suture  (N=60) | p-value |
| Antibiotic prophylaxis | 47 (79.7%) | 47 (78.3%) | 0.86 |
| Antibiotic therapy | 57 (96.6%) | 52 (86.7%) | 0.05 |
| Peritonitis | 46 (78.0%) | 49 (81.7%) | 0.63 |
| Main infectious focus  Gastric perforation (main focus)  Duodenal perforation (main focus)  Colon/rectum perforation (main focus)  Appendix perforation (main focus)  Intestinal ischemia (main focus)  Small bowel obstruction (main focus)  Tumor perforation (main focus)  Gynecological focus (main focus)  Other focus (main focus) | 14 (23.7%)  4 (6.8%)  24 (40.7%)  3 (5.1%)  3 (5.1%)  3 (5.1%)  3 (5.1%)  0 (0.0%)  4 (6.8%) | 9 (15.0%)  3 (5.0%)  23 (38.3%)  5 (8.3%)  8 (13.3%)  1 (1.7%)  2 (3.3%)  1 (1.7%)  7 (11.7%) | 0.23  0.68  0.57  0.78  0.10  0.30  0.20  0.32  0.41 |
| Drainage placed | 57 (96.6%) | 59 (98.3%) | 0.50 |
| Successful source control | 57 (96.6%) | 59 (98.3%) | 0.55 |
| Resection + stoma | 14 (24.1%) | 14 (25.0%) | 0.32 |
| Resection + anastomosis | 20 (34.5%) | 25 (44.6%) | 0.32 |
| Abdominal lavage | 58 (98.3%) | 59 (98.3%) | 0.99 |
| Surgical expertise/number of abdominal wall closures | 393.3 +/-679.2 | 392.5 +/-529.0 | 1.00 |
| Board certified surgeon | 53 (89.8%) | 54 (91.5%) | 0.75 |
| Teaching surgery | 12 (20.3%) | 11 (18.6%) | 0.82 |
| Data are means (with standard deviations) or numbers of patients (with percentages), p values are reported according to χ2 test for categorical variables and t-test for continuous variables. | | | |

| Table S2: SF36 Data 30 days after emergency laparotomy (Visit 4). | | | | |
| --- | --- | --- | --- | --- |
|  | Continuous group | Interrupted group | Total | p-value* |
|  | N=59 | N=60 | N=119 |  |
| physical functioning (0-100) | | | | 0.12 |
| - N  - Mean +/- SD | 30  54.2 +/-31.4 | 32  41.9 +/-29.5 | 62  47.8 +/-30.8 |  |
| role limitations because of physical health problems (0-100) | | | | 0.12 |
| - N  - Mean +/- SD | 30  30.0 +/-43.3 | 32  14.8 +/-30.4 | 62  22.2 +/-37.6 |  |
| bodily pain (0-100) | | | | 0.24 |
| - N  - Mean +/- SD | 30  58.6 +/-26.8 | 32  50.0 +/-30.3 | 62  54.2 +/-28.7 |  |
| general health perceptions (0-100) | | | | 0.61 |
| - N  - Mean +/- SD | 30  57.4 +/-21.6 | 32  54.8 +/-18.8 | 62  56.1 +/-20.1 |  |
| vitality (energy/fatigue) (0-100) | | | | 0.15 |
| - N  - Mean +/- SD | 30  51.0 +/-22.9 | 32  42.7 +/-22.0 | 62  46.7 +/-22.7 |  |
| social functioning (0-100) | | | | 1.00 |
| - N  - Mean +/- SD | 30  63.3 +/-35.5 | 32  63.3 +/-32.1 | 62  63.3 +/-33.5 |  |
| role limitations because of emotional problems (0-100) | | | | 0.90 |
| - N  - Mean +/- SD | 30  55.6 +/-45.8 | 32  54.2 +/-44.6 | 62  54.8 +/-44.8 |  |
| general mental health (0-100) | | | | 0.47 |
| - N  - Mean +/- SD | 30  68.0 +/-23.3 | 32  63.6 +/-23.6 | 62  65.7 +/-23.4 |  |
| Change in general health compared to last year  much better  a bit better  almost the same  a bit worse  much worse  missing | 2 (6.7%)  3 (10.0%)  7 (23.3%)  9 (30.0%)  9 (30.0%)  29 | 0 (0.0%)  1 (3.1%)  6 (18.8%)  12 (37.5%)  13 (40.6%)  28 | 2 (3.2%)  4 (6.5%)  13 (21.0%)  21 (33.9%)  22 (35.5%)  57 | 0.38 |
| standard. physical functioning scale | | | | 0.06 |
| - N  - Mean +/- SD | 30  37.2 +/-10.9 | 32  32.4 +/-9.4 | 62  34.7 +/-10.3 |  |
| standard. mental health scale | | | | 0.97 |
| - N  - Mean +/- SD | 30  46.9 +/-13.3 | 32  46.8 +/-12.5 | 62  46.9 +/-12.8 |  |
| *: p values are reported according to χ2 test for categorical variables and t-test for continuous variables. | | | | |

| Table S3: SF36 Data 12 months after emergency laparotomy Visit 5 (FAS) | | | | |
| --- | --- | --- | --- | --- |
|  | Continuous group | Interrupted group | Total | p-value* |
|  | N=59 | N=60 | N=119 |  |
| physical functioning (0-100) | | | | 0.17 |
| - N  - Mean +/- SD | 27  73.7 +/-28.4 | 26  61.7 +/-34.6 | 53  67.8 +/-31.9 |  |
| role limitations because of physical health problems (0-100) | | | | 0.14 |
| - N  - Mean +/- SD | 27  66.7 +/-43.3 | 26  48.1 +/-46.9 | 53  57.5 +/-45.6 |  |
| bodily pain (0-100) | | | | 0.34 |
| - N  - Mean +/- SD | 27  76.8 +/-32.8 | 26  68.7 +/-27.7 | 53  72.8 +/-30.4 |  |
| general health perceptions (0-100) | | | | 0.59 |
| - N  - Mean +/- SD | 27  66.1 +/-21.9 | 26  63.0 +/-19.9 | 53  64.6 +/-20.8 |  |
| vitality (energy/fatigue) (0-100) | | | | 0.77 |
| - N  - Mean +/- SD | 27  59.1 +/-23.5 | 26  57.1 +/-24.4 | 53  58.1 +/-23.7 |  |
| social functioning (0-100) | | | | 0.16 |
| - N  - Mean +/- SD | 27  85.2 +/-19.3 | 26  76.4 +/-25.3 | 53  80.9 +/-22.7 |  |
| role limitations because of emotional problems (0-100) | | | | 0.09 |
| - N  - Mean +/- SD | 27  88.9 +/-27.7 | 26  71.8 +/-41.8 | 53  80.5 +/-36.1 |  |
| general mental health (0-100) | | | | 0.27 |
| - N  - Mean +/- SD | 27  78.1 +/-16.9 | 26  72.2 +/-21.4 | 53  75.2 +/-19.3 |  |
| Change in general health compared to last year  much better  a bit better  almost the same  a bit worse  much worse  missing | 8 (29.6%)  7 (25.9%)  6 (22.2%)  4 (14.8%)  2 (7.4%)  32 | 12 (46.2%)  5 (19.2%)  6 (23.1%)  2 (7.7%)  1 (3.8%)  34 | 20 (37.7%)  12 (22.6%)  12 (22.6%)  6 (11.3%)  3 (5.7%)  66 | 0.72 |
| standard. physical functioning scale | | | | 0.29 |
| - N  - Mean +/- SD | 27  45.0 +/-13.1 | 26  41.1 +/-13.3 | 53  43.1 +/-13.2 |  |
| standard. mental health scale | | | | 0.29 |
| - N  - Mean +/- SD | 27  53.4 +/-7.7 | 26  50.6 +/-11.5 | 53  52.0 +/-9.8 |  |
| *:p values are reported according to χ2 test for categorical variables and t-test for continuous variables. | | | | |

Fig. S1: Handling of missing follow up visits.


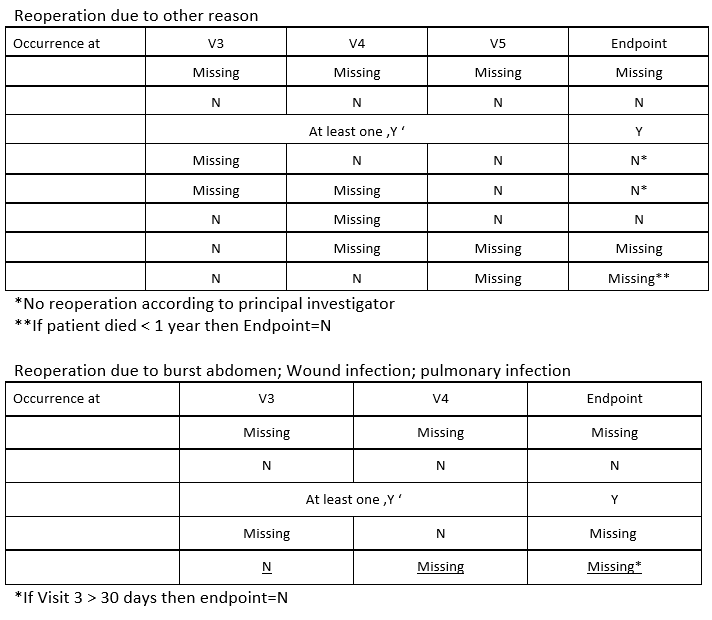


| Abbreviations. Y: A follow-up visit has been documented, N: No follow-up visit documented |
| --- |
